# Supporting instructional change in mathematics: using social network analysis to understand online support processes following professional development workshops

**DOI:** 10.1186/s40594-018-0120-9

**Published:** 2018-07-02

**Authors:** Charles N. Hayward, Sandra L. Laursen

**Affiliations:** Ethnography and Evaluation Research, University of Colorado Boulder, 580 UCB, Boulder, CO 80309 USA

**Keywords:** Inquiry-based learning, Pedagogy, Instructional change, Professional development, Online

## Abstract

**Background:**

Studies continually show benefits of active learning in college classrooms, yet it is difficult to get faculty to adopt these methods. Particularly challenging is the final step of the instructional change process, “refreezing,” when after making initial changes in instructional methods, instructors decide whether to continue with new instructional methods or return to their previous methods. Though this stage is important, it is not well studied. Most available studies about ongoing support following professional development on teaching merely state that facilitators made an effort to offer support, or report how frequently participants engaged with online support mechanisms through counting postings on listservs or message boards. Such measures do not show evidence that participants actually received positive reinforcement or intellectual and emotional support, which are crucial to refreezing, nor do these frequency analyses help other professional developers learn how to create productive ongoing support mechanisms that yield high participant engagement.

**Results:**

This workshop for 35 college mathematics instructors used online and in-person communities to provide support to participants during the post-workshop period of “refreezing.” Almost all workshop attendees participated in “e-mentoring” (94%), primarily through a productive, engaging group email listserv. By combining qualitative coding of message content with the techniques of social network analysis, we reveal how facilitators and participants on the group listserv provided intellectual and emotional support, as well as positive reinforcement through feedback loops. The analysis also shows how the facilitators made this a helpful group and maintained participant engagement through frequent encouragement, deliberate community building, and thoughtfully timed responses.

**Conclusions:**

Though many professional development workshops offer online support through email listservs, there is little evidence that these listservs successfully engage and support participants. Applying the analytic approach of social network analysis allowed us to model the conversation threads in one highly engaged and supportive listserv following a mathematics professional development workshop. This method revealed the processes of ongoing support in ways that traditional frequency-based analyses cannot. This method also revealed lessons for how other professional developers can create productive, helpful online support listservs. Since this is an innovative application of social network analysis, we describe the method in detail.

**Electronic supplementary material:**

The online version of this article (10.1186/s40594-018-0120-9) contains supplementary material, which is available to authorized users.

## Background

Numerous studies have found benefits to undergraduate students from the use of active learning methods in science, technology, engineering, and mathematics (STEM) fields (Freeman et al. 2014). According to Freeman et al. (2014), the benefits are so strong that “If the experiments analyzed here had been conducted as randomized controlled trials of medical interventions, they may have been stopped for benefit—meaning that enrolling patients in the control condition might be discontinued because the treatment being tested was clearly more beneficial” (p. 4). While the evidence to support use of active learning strategies is strong, getting large numbers of faculty to adopt these new methods is difficult (Fairweather 2008; Henderson and Dancy 2007, 2008, 2011). Professional development (PD) workshops are one strategy for helping college instructors to adopt research-supported teaching methods. Workshops are the preferred method of National Science Foundation (NSF) program directors, particularly when they are multi-day, immersive workshops and include follow-up interaction between participants and facilitators (Khatri et al. 2013).

Other authors extol the virtues of online interactions for professional development in higher education. Bierema and Merriam (2002) pointed out the advantages of “e-mentoring” as “boundaryless” and “egalitarian” since it “has the potential to cross barriers of race, gender, geography, age, and hierarchy that are rarely crossed in traditional mentoring relationships” (p. 220). Brooks (2010) theorized that “hybrid” faculty development blending online and in-person components may be more effective than solely in-person approaches, since it would mirror the reality of many hybridized college courses and because online faculty development may have advantages over face-to-face faculty development for people with marginalized backgrounds. Fairweather (2008) argued that successful change strategies for improving STEM education might involve “external networks of like-minded colleagues… [who] can be important forces in promoting instructional reform” (p. 17). Gunawardena et al. (2009) proposed a theoretical framework for building online communities of practice and suggested that discussions in online peer groups more naturally operate within Vygotsky’s classic zone of proximal development (Vygotsky 1978), whereas expert/novice relationships may be more mismatched.

The recommendations of these theoretical pieces bear out in practice: successful professional development programs in higher education include sustained peer interaction over a long duration—often weeks or months (Giersch and McMartin 2014). This presents a challenge for one-off PD workshops, so many discipline-based PD workshops in STEM higher education often plan follow-up interaction through group email lists, reunion meetings, and mentoring (Council of Scientific Society Presidents 2012). However, among studies of professional development workshops offered in biology, chemistry, engineering, geosciences, mathematics, microbiology, and physics/astronomy, there is little available research about these follow-up components beyond short descriptions of the follow-up activities that were provided (Council of Scientific Society Presidents 2012). For example, following workshops for new chemistry faculty, facilitators created monthly online discussion sections in order to “provide a trusted community for faculty who may [have] no other support” (Stains et al. 2015, p. 1474). The authors reported only a few topics that participants discussed. In our earlier study about a set of three workshops for college math instructors, only one set of workshop leaders successfully implemented their plan to engage participants in an email group after the in-person workshop (Hayward, Kogan, and Laursen 2016). Because they were not a routine feature of the workshops, we did not deeply analyze those follow-up activities.

Among studies that do provide evidence about participant engagement with online follow-up mechanisms, most simply offer descriptive statistics such as participation rates and frequency measures. For a physics faculty workshop, evaluators counted various activities in a follow-up online discussion forum, such as starting a new discussion topic or replying to an ongoing discussion topic (Corbo et al. 2016). However, these measures describe only frequencies of participation and do not necessarily show that participants’ activity supports their professional development or instructional change nor do such measures offer insight on how to sustain participant engagement: many professional developers create online follow-up components, but it is not given that participants will engage with them.

Others have attempted to provide insight from qualitative analysis into how participants use online resources. Vaughn and Garrison (2006) compared how participants interacted in online and in-person PD components by pairing qualitative analysis of online discussion transcripts with participant interviews. This approach offered a richer picture than simple descriptive statistics but required significant effort to create and code transcripts and conduct interviews.

Collectively, this literature identifies a gap in our understanding of follow-up practices for professional development in STEM higher education. Just as effective physical therapy is necessary to realize the best outcomes from knee replacement surgery, effective follow-up is needed to cement the instructional changes encouraged by a professional development workshop. Yet the physical therapist uses and refines practices distinct from those of the orthopedic surgeon, and likewise, the follow-up activities are distinct from the workshop; they can be separately studied and improved. In this paper, we examine follow-up support using data collected following an intensive, week-long professional development workshop for college mathematics educators on inquiry-based learning (IBL). Throughout the year after the workshop, facilitators engaged most participants in online follow-up through a group listserv as they sought to implement these methods in their own courses. We have separately detailed workshop design and outcomes and how these align with prior literature on professional development (Hayward, Kogan, and Laursen 2016); here we focus on the follow-up phase itself. We apply an innovative approach for analyzing participant engagement in this email listserv by marrying traditional qualitative coding of message content with the quantitative, relational techniques of social network analysis (SNA). Through this approach, we gain deeper insight into the processes of support and offer some lessons about how facilitators can create and sustain a supportive online community.

## Conceptual framework

This paper draws on two bodies of knowledge, instructional change theory and social network theory. We use the same three-stage model of instructional change that we previously used to understand and interpret IBL workshops (Hayward, Kogan, and Laursen 2016). Developed by Lewin in 1947, the model includes the stages of (1) *unfreezing*, (2) *changing*, and (3) *refreezing*. Paulsen and Feldman (1995) adapted this general model of system change to describe instructional change; it is advantageous in accommodating not only the conceptual shifts involved in an individual instructor’s path to change but the potential influences of external stakeholders and cultures. This model is widely cited in higher education literature on instruction and has been previously applied in empirical studies of professional development for STEM instructors (Andersen et al. 2016; Connolly and Millar 2006; Sirum and Madigan 2010; Nadelson et al. 2013). Nonetheless, most existing studies of professional development in higher education (summarized by the Council of Scientific Society Presidents 2012) have not been theoretically driven. In Giersch and McMartin’s (2014) review of PD programs in higher education, they point out that studies rarely consider the theory of instructional change and tend to simply report “how we did it good” (p. 5), describing immediate changes in attitudes or beliefs with little consideration of follow-up support or long-term outcomes. Even in K-12 educational literature, where professional development has been much more studied, there is no common conceptual framework for professional development and instructional change (Desimone 2009).

We have previously applied Paulsen and Feldman’s framework to provide a detailed analysis of how IBL workshops helped participants through the first two stages (Hayward, Kogan, and Laursen 2016). In that paper, we placed our results in context with the existing literature on designing and implementing high-quality professional development, which emphasizes the importance of active participation, sufficient time to explore and revisit topics, and addressing common participant concerns about active learning strategies, such as student resistance, content coverage, and instructor skills for implementation. In this report, we describe these first two stages briefly in order to provide context for the third stage, *refreezing*, that is the focus of this study.

During *unfreezing*, instructors gain motivation to change through experiencing incongruence of their teaching goals with the outcomes of their teaching practices. In the next stage, *changing*, instructors learn, apply, and reflect on new teaching strategies so that their behaviors work toward their desired outcomes. In this stage, teaching strategies are fluid as instructors test and refine new methods. In our previous paper (Hayward, Kogan, and Laursen 2016), we offered evidence from surveys and interviews that instructors did change their instruction in the direction espoused by the workshop. Based on workshop observations, we also argued that the design, conduct, and overarching messages of this workshop series were instrumental in facilitating the *unfreezing* and *changing* stages. For example, the workshop’s attention to how IBL strategies could be adapted for different settings helped instructors find ways to teach that were consistent with their own identities and classroom contexts, thus aiding *unfreezing* and providing a viable, individualized vision of how to change. The workshops were designed to help with *changin*g by incorporating features of effective professional development including active participation and addressing common participant concerns.

In the final stage, *refreezing*, either the new strategies are “reconfirmed” and solidified through positive feedback and support or the instructors return to their original strategies. When instructors find versions of IBL that work for them and their students, they may *refreeze* as an IBL instructor. If they are unable to find a version that works well, they may return to the methods they used before the workshop. In Lewin’s (1947) original paper, he presents evidence that changes initiated during participatory group discussions are larger and more lasting than changes initiated following lectures or individual decisions. This is particularly important for our current context of group workshops and listservs.

Among existing studies of effective professional development workshops in STEM higher education, stage two, *changing*, is the most studied of the three stages. The Council of Scientific Society Presidents (2012) summarized results of existing professional development programs in STEM and noted that most programs do not even have sufficient evidence of effective *changing* because they often do not measure outcomes beyond participant satisfaction with the PD. Some researchers have studied *changing* through observing shifts in instructors’ teaching practices following professional development, with mixed results (e.g., Derting et al. 2016; Ebert-May et al. 2011; Stains et al. 2015). These researchers acknowledge that long-term changes should be measured beyond just the first year or two following a PD experience (Derting et al. 2016; Stains et al. 2015). Overall, evidence is limited on the effectiveness of PD in generating change for postsecondary instructors and on whether those changes are sustained long-term—and there is even less on the processes of sustaining those changes, *refreezing* (Connolly and Millar 2006).

Our focus throughout this paper is specifically on the role of follow-up support in solidifying and securing instructional change, *refreezing*—not on the efficacy of the workshops themselves nor the longevity of instructional changes. To return to the prior metaphor, in this analysis, we are examining the practices of the physical therapy following surgery, not the knee replacement procedure itself. In a previous paper, we provided evidence from surveys and interviews about how IBL workshops supported participants through the first and second stages, *unfreezing* and *changing* (Hayward, Kogan, and Laursen 2016), but did not have sufficient evidence at that time to explore the *refreezing* stage. Here we focus on the third stage of Lewin’s model, *refreezing*. Follow-up support is challenging when workshop participants are geographically scattered and cannot easily reconnect face to face, but facilitators of the workshop we studied leveraged other IBL-related resources and events and created a group email list to help workshop participants through *refreezing* by providing the ongoing support, positive feedback, and confirmation essential to this stage.

To study *refreezing*, we take two complementary approaches. First, we use a mix of surveys and frequency analyses of email listserv activity to explore the following research questions:

1. What resources can workshop leaders use to support instructors through refreezing?
2. How can instructors’ participation in online support activities be optimized?

However, these data sources and questions offer only a partial view of the *refreezing* process. Participation in online support activities is largely about relationships, yet relationships are difficult to study through surveys and frequency analyses. Social network analysis (SNA), on the other hand, is a method developed specifically for studying relations among actors (Grunspan et al. 2014). Based on mathematical graph theory, SNA is a way of visualizing and analyzing networks of relationships or connections (represented as edges or lines) formed among actors (nodes or dots) (Butts 2008). Both nodes and edges have attributes or qualities, so with SNA, it is possible to analyze how characteristics of actors affect relationships or how characteristics of relationships affect actors. SNA has been used for diverse purposes including analyzing how the structure of relationships affects social capital (Granovetter 1973), how relationships form based on demographic similarities (McPherson et al. 2001), and how friendship networks contribute to the spread of obesity (Christakis and Fowler 2007). In many examples of SNA, the nodes or actors in the network are people, though they could also be organizations (e.g., Stuart et al. 1999) or other interrelated entities. The edges represent some sort of connection between nodes, and in higher education, these relationships could be co-authorship or working relationships such “discussing teaching” together (e.g., Quardokus and Henderson 2015). In some cases, edges are directional and represented with arrows. For example, in a pair, if person A reports that person B is a friend, the arrow would point from A to B. If both people report friendship, then the edge between A and B representing their relationship is reciprocal and shows arrowheads at both ends.

By using SNA methods to study patterns of conversations in a group email list, we can explore research question 2 more deeply, in ways that frequency analyses do not allow. Since SNA records relationships that can be analyzed based on qualitative characteristics of nodes and edges, we can look at how conversations flowed on the listserv based on characteristics of messages or senders. Therefore, we can explore a third research question:

(3) How can group email lists support refreezing through positive feedback and emotional and intellectual support?

## Methods

In this section, we discuss the context for this study. Then we describe the research methods, first surveys, then listserv analysis using both frequency and social network techniques. Prior to any data collection, the study was reviewed by our university’s Institutional Review Board.

### Study context

We provide some background information to help understand the workshop itself, before tightening our focus on how to support participants following a workshop. Previously published works describe the IBL workshops, detail the methods used to document workshop delivery and outcomes, and lay out evidence that workshops were effective in supporting participants through the unfreezing and changing stages (Hayward, Kogan, and Laursen 2016; Hayward and Laursen 2016). Forthcoming work from our research group will provide additional evidence about the validity of self-reported teaching practices to measure change following workshops.

The ultimate goal and measure of success of PD workshops is improved student learning, yet this outcome lies far downstream from the workshop (Guskey 2002). Measuring student learning is not always feasible due to cost and complexity that are beyond the scope and budget of many projects. Therefore, rather than measuring student outcomes directly, we use a “golden spike” approach (Brown Urban and Trochim 2009), linking instructors’ use of IBL practices to other research showing that classroom use of IBL practices is linked to improved student outcomes, especially for women and low-performing students (Kogan and Laursen 2014).

Our analysis derives from a larger study of four workshops on inquiry-based learning (IBL) for college mathematics instructors held 2013–2015. IBL is a form of active learning in college mathematics emphasizing practices that share the spirit of student inquiry through the core features of (1) deep engagement with rich mathematics and (2) collaboration with peers (Yoshinobu and Jones 2013). Other sources offer more detailed descriptions of IBL (e.g., Ernst et al. 2017).

Each workshop was 4 days long and featured a mix of sessions designed to help participants learn about IBL and prepare to implement it in their classrooms. Four types of sessions all featured active participation and self-reflection, which are characteristics of effective professional development (Cormas and Barufaldi 2011; Garet et al. 2001). The sessions were (1) reading sessions to discuss research articles, (2) video sessions to watch and analyze IBL classes, (3) nuts and bolts sessions to develop plans for how to structure and run an IBL class, and (4) course content sessions for participants to work in small groups to develop IBL materials for their own courses.

Facilitators created a closed, unmoderated email listserv for each workshop cohort. Any message from pre-approved senders—participants, facilitators, and evaluators—was immediately distributed to all list members. At the workshop, participants consented to have the content of their emails included in our network data set. Over the course of the year following the workshop, we observed the post-workshop listserv conversations; cataloged messages with identifying information such as the time, date, and sender; and tracked the number of messages sent per participant. We also coded “facilitator prompts,” meaning an invitation from one of the workshop leaders for participants to “chime in” or send a message to the list.

### Survey methods

Separately, for all four workshops, we collected surveys pre-workshop, post-workshop, and one academic year later. The surveys included all of the same items as workshop surveys published previously (Hayward and Laursen 2014, 2016), which assessed beliefs about IBL, teaching practices, feedback on the workshop, and demographic information. Some items were designed to address the *refreezing* process through assessing participants’ attendance at IBL-related events and engagement with support resources.

Data presented here come from the second of the four workshops, though all were very similar in design and immediate outcomes (Hayward and Laursen 2016), and post-workshop listserv cohorts functioned similarly. Quantitative survey data were analyzed using SPSS v. 22 (IBM Corp 2013), and text items were coded in Microsoft Excel (Microsoft 2011).

### Listserv analysis

In order to assess participants’ engagement in the ongoing group email list, we first conducted a frequency analysis, then conducted qualitative coding of the types of messages sent on the list and analyzed the patterns of communication with SNA methods.

#### Frequency analysis

We tracked and coded all listserv messages, entering each into the database with identifiers including time, date, sender, and subject line. We also recorded whether it came from a new (first-time) or repeat sender on the list and whether it was from a participant or facilitator. For each facilitator message, we coded whether it invited or prompted participants to engage—not simply a question participants could respond to but an open invitation such as “we’d like to hear from you” or “let us know what you’re up to.”

#### Social network analysis methods

Throughout the year, as we read and discussed the content and tone of listserv conversations, we noticed that feedback loops sometimes developed: participants might raise a concern to the group, others would provide suggestions, and then the original participant would report back after testing out the collegial suggestions. We also noticed a surprising degree of emotion in the messages being exchanged. For instance, when a participant reported feeling discouraged by their students’ level of engagement, others responded not only with ideas but also with emotional support and encouragement. Such social exchanges prompted our interest in not just the frequency but the content of listserv messages. We realized that social network analysis (SNA) methods might best capture the evolving nature of connected conversations in an email thread.

**Fig. 1:**
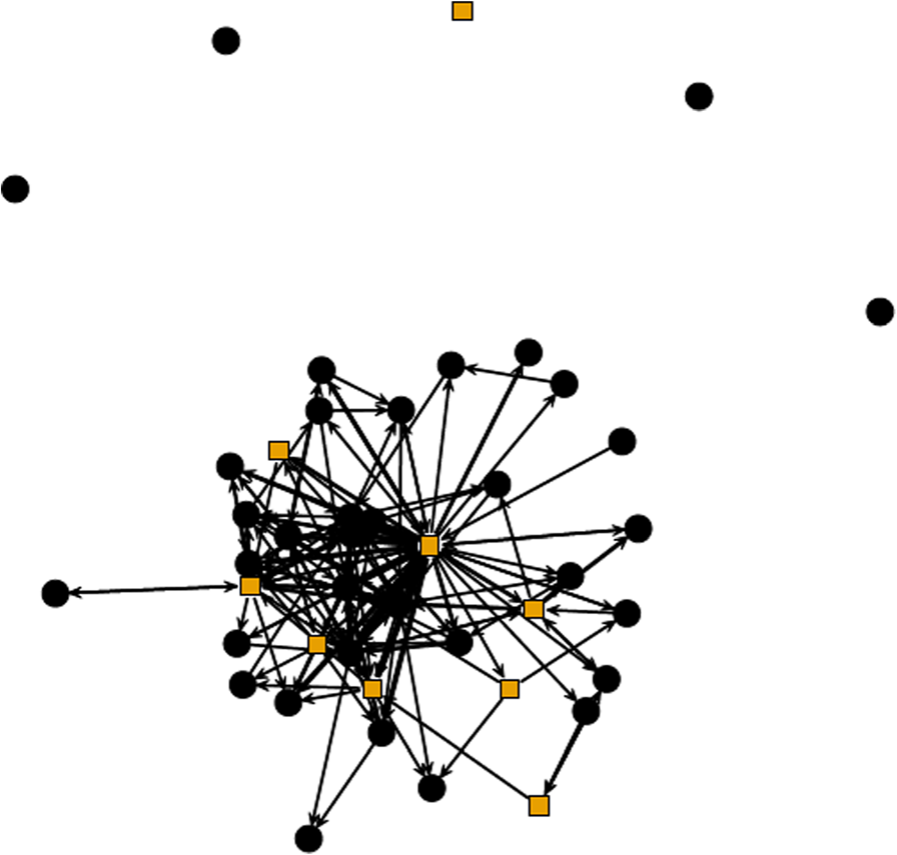
Network diagram of connections between participants (dark circles) and facilitators (light squares) on group listserv

A simple social network diagram (Fig. 1) shows the emails exchanged on the workshop listserv where nodes represent individuals and edges represent messages sent between them. The light-colored, square nodes represent workshop facilitators, and the dark, circular nodes represent participants. Here, arrows originate from the message sender and point towards the individual to whom the message responded; they are bidirectional when both senders sent messages responding to each other. The line widths of edges are wider when more messages were exchanged between specific pairs of individuals. This network treating people as nodes is not very informative. It makes clear that five individuals did not interact on the list and that some interacted with more people than others, but otherwise, it is difficult to draw meaningful conclusions.

Instead, we treated the email messages themselves as the nodes in a network. This way, we can examine connections between types of messages, model conversation threads, and understand how support processes develop over time. SNA is well suited for studying relational data in the context of educational improvement (Daly 2012), and emails are inherently relational and social. Since SNA allows for directionality of relationships, it captures the patterns of response between messages in conversation threads. A listserv is a good source of easy-to-obtain network data, since the listserv is closed and bounded (Grunspan et al. 2014), meaning it contains all possible network data. While the listserv itself is bounded, our analyses cannot track or account for additional private conversations outside the listserv.

**Fig. 2:**
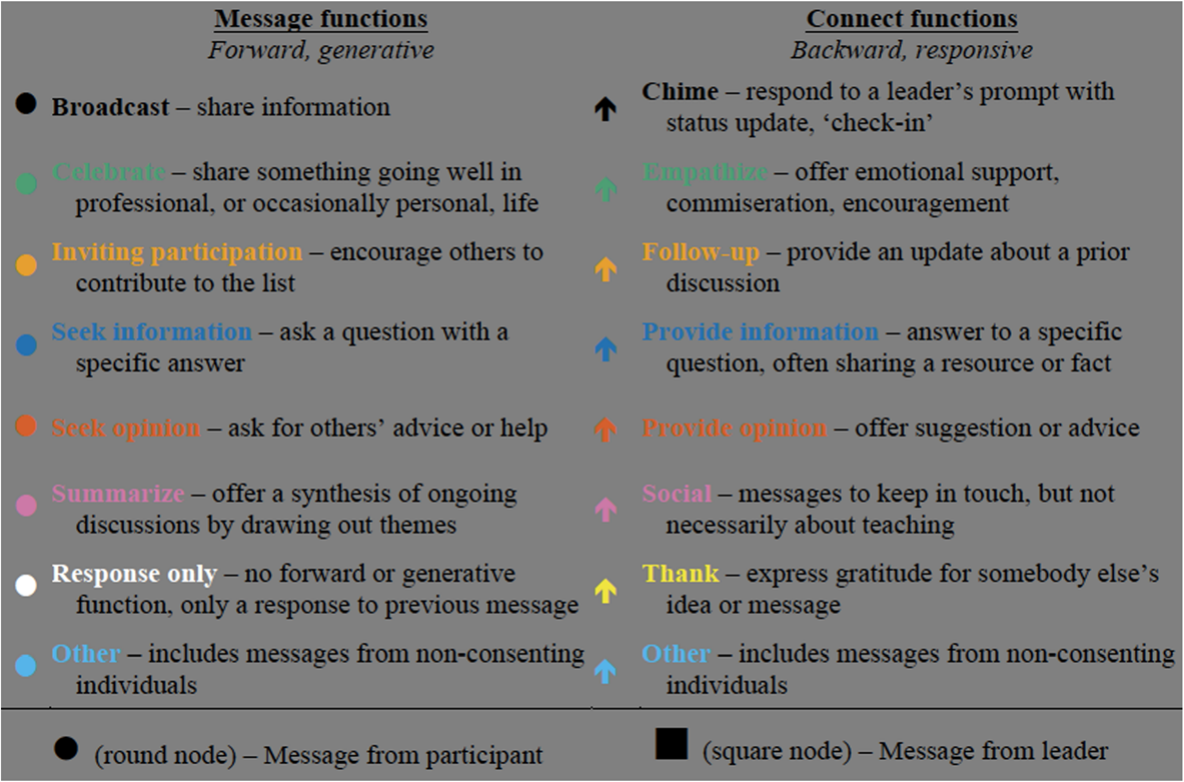
Common key of m*essage function* and c*onnect function* categories for all network figures

SNA analysis uses both quantitative and qualitative data. In this study, the network is made of messages (nodes) connected by responses (edges) and can be quantitatively analyzed using methods from graph theory (Butts 2008). When paired with qualitative coding of node and edge characteristics, meaningful patterns can emerge. We developed a qualitative coding scheme that attended to message characteristics in two ways: *message functions* are forward-looking (generative) and relate to how a message invites further conversation*.* They are therefore represented as characteristics of nodes. *Connect functions* are backward-looking (responsive) and relate to how a message connects to previous messages. Connect functions are therefore represented as characteristic of the edges between message nodes in our network. Rather than relying solely on subject lines of messages, we manually coded all connections by determining what earlier message(s) each message referred to based on the topic, quoted replies, or by specific references to earlier messages or individuals. We developed coding categories for generative (message) and responsive (connect) functions through the process of constant comparative coding (Glaser 1965), as detailed in Fig. 2, which also shows the color-coded symbols used consistently in network figures throughout this paper. The color palette was developed so that individuals with the most common types of color blindness can still distinguish the various hues (Okabe and Ito 2008).

Each message was coded with only one main *message function*. If the message responded to any previous message, *connect functions* were separately coded, such that each message could have multiple *connect functions*. For example, if a participant thanked three people who had earlier offered ideas, three yellow arrows are drawn from this node, one to each of the three prior messages. However, between any pair of messages, only one *connect function* was possible.

Exchanges coded as information tended to reflect specific, question/answer interactions (e.g., “Where can I find an IBL textbook for linear algebra?”) whereas opinion exchanges tended to invoke broader topics and multiple viewpoints shared in discussions (e.g., “How do I get my students to participate in class?”). For both *message* and *connect functions*, “other” codes included messages that did not fit the defined categories and those from a few individuals who did not give permission to include the content of their messages in the database. Use of identifying information for these messages (time/date/thread) maintained the structure of email communications, so that the network structures did not appear as disconnected discussions.

In addition to the primary *message* and *connect functions* described above, some ancillary functions were separately coded that could occur in conjunction with any primary function. For example, a participant may have responded to another’s concern, “Whoa, that is a difficult situation!” followed by an opinion about how to address the concern. The primary *connect function* of such a message is “provide opinion”; to also capture that emotional support was offered, we separately coded the ancillary function, “community building.” Community building included all messages coded as “celebrate,” “friendship,” “thank,” and “emotional support,” as well as other types of messages that included such expressions even if not their main function.

Similarly, each message was coded for “facilitator prompting,” which could also be a primary or ancillary function. Topical questions were considered “facilitator prompting” only when the message also included an explicit, general invitation such as “Chime in and give us an update!” Finally, messages and connections were identified and sorted by secondary characteristics including sender role (facilitator or participant) or first-time message (the first message each individual sent on the group list, regardless of function). From the coded data, we created social network diagrams and carried out analyses in *R* (R Core Team 2016) using the packages *sna* (Butts 2016), *igraph* (Csardi and Nepusz 2006), and *intergraph* (Bojanowski 2015). We tested for differences between types of messages using *t* tests and Mann-Whitney tests, as appropriate, in Excel (Microsoft 2011) and SPSS v. 22 (IBM Corp 2013).

## Results

We first present survey results to highlight participants’ engagement with post-workshop support resources. Then we present listserv analyses, first frequency analysis, and then social network analysis. Frequency analysis shows how often participants and facilitators engaged in the email list. Social network analysis gives more detail about how participants and facilitators interacted on the list and how this supported participants emotionally and intellectually.

### Survey results

The workshop served 35 participants. Of these, 54% identified as women and 43% as men (3% did not respond). They represented varied career stages and institution types, though most were untenured faculty (54%) and came from 4-year colleges (69%). Most were early in their careers with 2–5 years of teaching experience (51%) or less than 2 years (17%). All 35 (100%) completed the pre- and post-workshop surveys and 28 (80%) completed the 1-year follow-up survey. Pre/post/follow-up surveys were matched by respondent using anonymous identifiers.

#### Participants use IBL

Though stage two, *changing*, is not the focus of this study, we first provide evidence that participants have progressed through this stage so that we can then focus on the next stage, *refreezing*. We assessed participants’ progress through *changing* by measuring implementation of IBL in multiple ways. On 1-year follow-up surveys, 26 (93%) of 28 respondents directly reported using IBL methods in their classes, ranging from “some IBL methods” (25%) to “more than one full course” (39%). Seven did not answer this survey, so the conservative estimate of IBL use is 26 of 35 workshop participants or 74%. In messages sent on the listserv, 30 of 35 participants (86%) made comments indicating that they were using IBL methods: they described using IBL-related practices or materials in their classrooms or asked questions about IBL-related practices, such as how to deal with student anxiety about presenting problem solutions. Combining these measures by individual reveals that at least 31 participants (89%) were using at least some IBL methods.

**Fig. 3:**
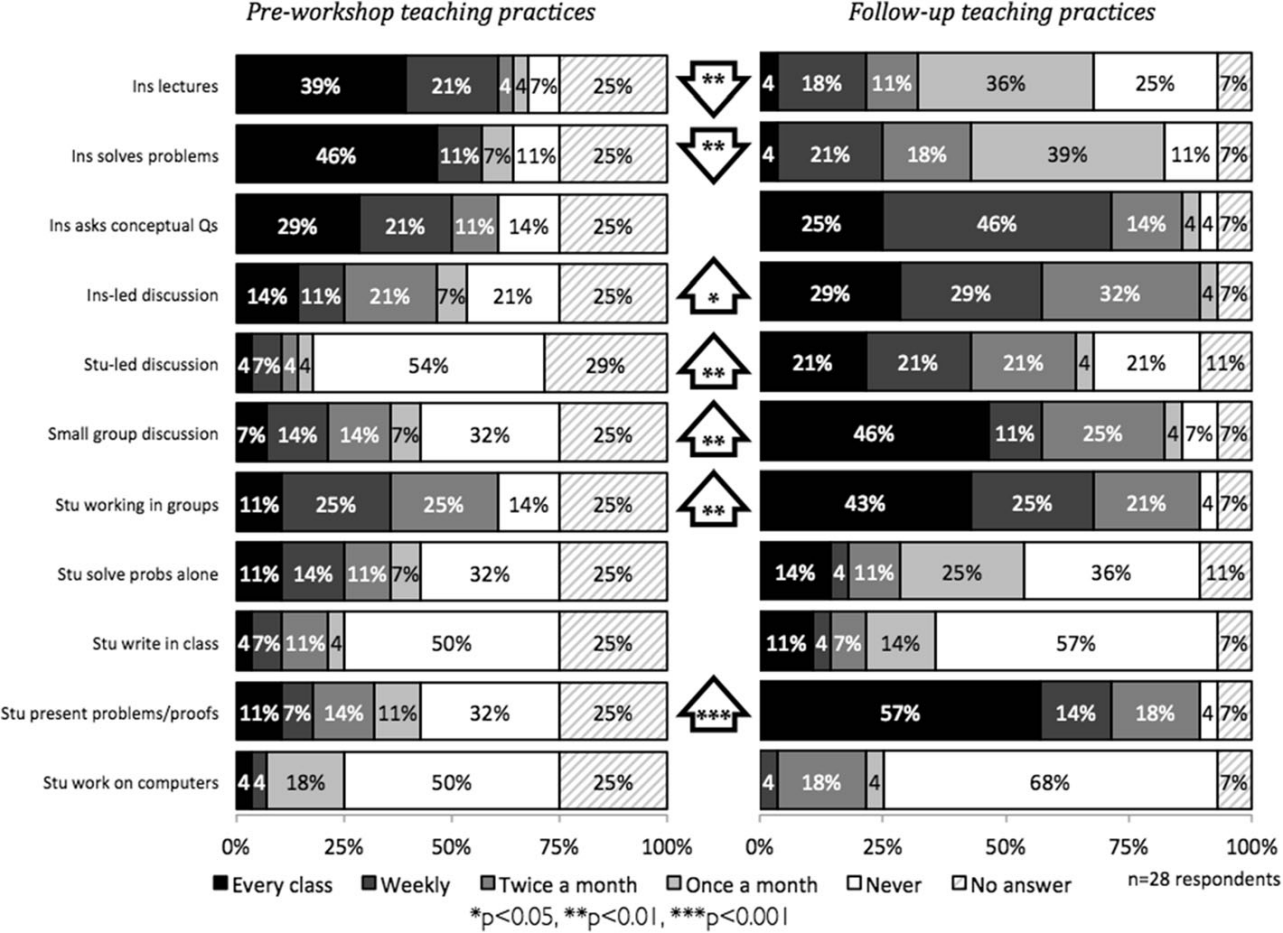
Changes in frequency of use of teaching practices from pre-workshop to 1-year follow up (*n* = 28 participants). (Adapted from Hayward, Kogan, and Laursen 2016)

We also measured IBL indirectly through documenting changes in reported teaching practices from pre-workshop to 1-year follow-up. These changes were consistent with IBL practice as well: decreases in instructor-led activities (lecture and solving problems) and increases in some student-centered activities (discussions, group work, presentations), as detailed in Fig. 3. Overall, these multiple data sources indicate high usage of IBL methods.

#### Follow-up activities and resources support implementation of IBL

High reported use of IBL methods indicates individuals moved through stage two, *changing*, of Paulsen and Feldman’s instructional change model. Stage three, *refreezing*, is characterized by sustained use of new instructional methods reinforced by positive, ongoing support and feedback. To assess support and feedback, we asked participants on follow-up surveys to report their engagement with various IBL-related events and resources that were available in the broader IBL community. It is also possible that participants may *refreeze* by returning to their original teaching methods. This type of *refreezing* is non-existent in our experience studying participants’ initial attempts to use IBL in the first year after the workshop. However, if these initial attempts to implement IBL are not successful, it is possible that over time, participants may return to their previous methods. Our research team is also conducting a long-term follow-up study with participants from similar IBL workshops held 7 to 11 years ago, in order to determine how frequently *refreezing* may involve returning to previous methods.

##### IBL events

Of the 28 respondents to the follow-up survey, 68% had participated in another IBL-related event in the year after the workshop. Events included IBL sessions at national and regional professional meetings, including the Joint Mathematics Meetings (JMM), Mathematical Association of America section meetings, MathFest, and the annual IBL conference. Some people also presented at these events (7, 25%). Altogether, 22 participants (79%) had attended or presented at another event in the first year. While some events were more popular than others, the variety of related events made it possible for many participants to engage in at least one.

##### IBL resources

Most respondents to the follow-up survey (25, 89%) reported using IBL resources, most frequently the workshop listserv (21, 75%) and the *Journal of Inquiry Based Learning in Mathematics* (12, 43%), a compendium of peer-reviewed problem sequences for specific IBL courses. A few had used the mentor program and mini-grants provided by the Academy of Inquiry Based Learning (AIBL). Again, participants engaged with a number of different resources, and the variety available may help to meet varying needs of participants.

##### IBL support

On the follow-up survey, we asked participants to report the level of support they received from their institutions. Overall, a majority felt supported and rated key institutional figures as “mostly supportive,” including department heads/chairs (64%) and departmental colleagues (50%). Some participants reported active support (12, 43%) through encouragement or financial support, while others reported support as the absence of obstacles—they were able to make decisions autonomously (5, 18%). Only a few reported feeling doubted or discouraged by colleagues (3, 11%). Thus, while collegial resistance was not a barrier for most, only 43% of respondents locally received the explicit and active support that is crucial to *refreezing*.

### Listserv analysis results

The group listserv was the most commonly used form of ongoing support during *refreezing*. We examine this activity closely in two ways. First, we present frequency counts and descriptive statistics of interaction on the listserv to reveal some features of the workshop and leaders’ work to encourage participation. Then, we present a social network analysis offering more nuance about how list conversations helped to support participants’ *refreezing*. This social network analysis looks at the relationships among messages and reveals how conversations flowed in the group listserv.

#### Frequency analysis

In similar, earlier workshops, efforts by workshop leaders to engage workshop participants in online follow-up were not successful (Hayward, Kogan, and Laursen 2016). However, all four workshops in the series studied here successfully engaged many attendees in online follow-up. We chose one mid-series workshop as a sort of “good case scenario”—leaders had a prior year of experience facilitating online e-mentoring but were also still learning. List members included all 35 participants and all 11 members of the facilitation team from the larger project, 8 of whom had been present at this particular workshop. Participation was high with 91% of attendees active in the listserv and 281 messages exchanged on the listserv.

##### Push technologies help

Social networking platforms like Facebook or Google+ seem to many a good way to keep in touch and are suggested at most workshops. One attendee from this workshop created a private group on a social networking platform, and 24 people (55%) from the workshop joined it. But only 4 people (9%) exchanged eight messages in the two days following the workshop; in the next two years, the group had no activity. In contrast, 32 (91%) of workshop participants were active on the official group listserv at some point in the year after the workshop. In the first year, they exchanged 281 messages, and the list remains active 2 years later. These observations suggest that “push” technologies, like email, that deliver messages directly may be more successful than those requiring participants to log in and seek them out.

##### Leaders promoted participation

**Fig. 4:**
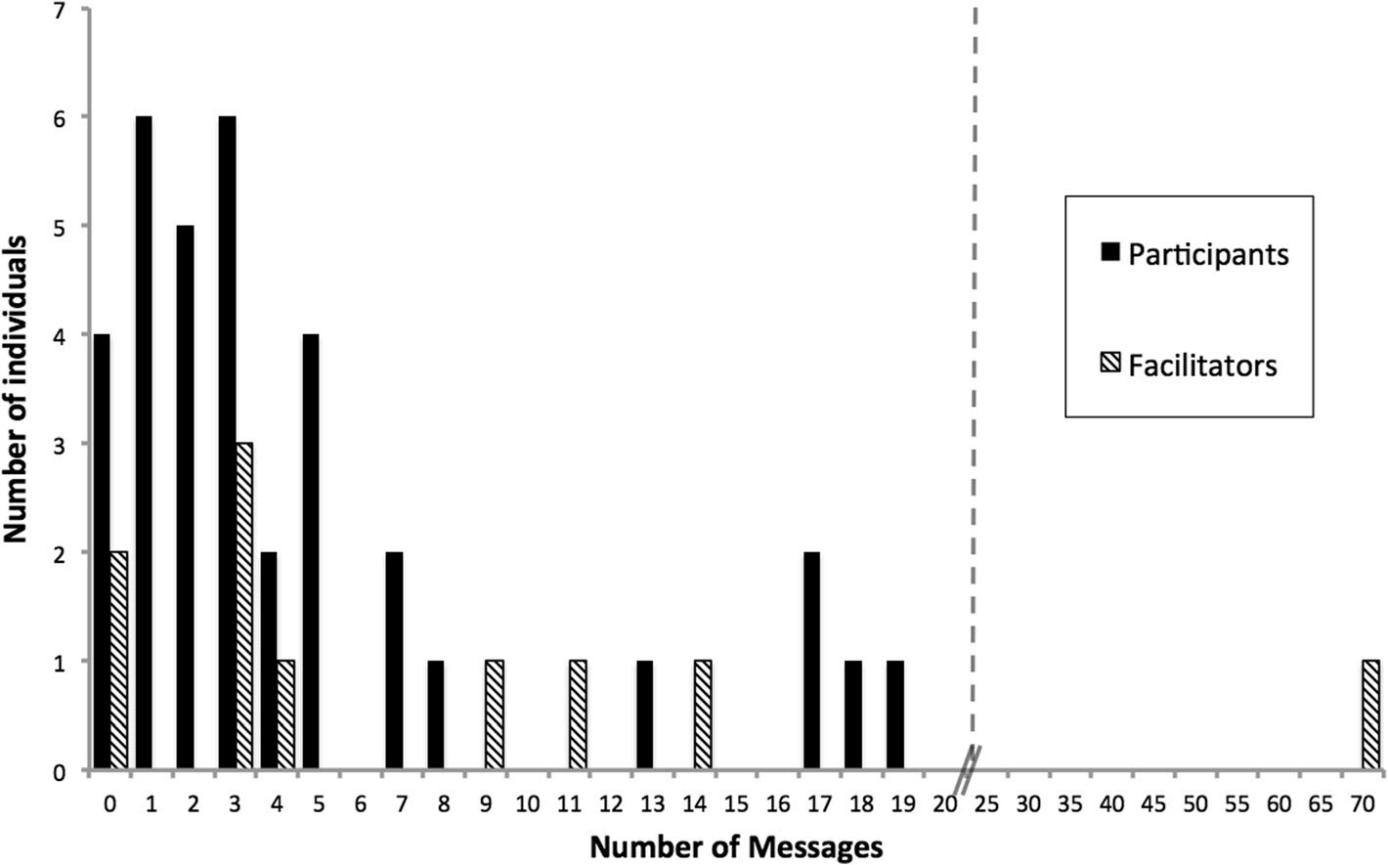
Frequencies of individuals’ activity on group email list (*n* = 46, 35 participants and 11 facilitators)

Facilitators sometimes prompted the list to generate discussions, but all members were free to send messages to the entire group at any time. The list was closed but unmoderated. Frequencies of individuals’ participation are shown in Fig. 4. Most individuals (27, 77%) sent five or fewer messages to the list in the year following the workshop. Leaders were more active; most sent 15 or fewer messages to the list over the year, but one facilitator sent 66 messages, almost a quarter of all messages on the list. Many messages announced upcoming events or resources, but others were prompts for interaction, inviting participants to share their current IBL teaching activities and raising topics for discussion.

**Fig. 5:**
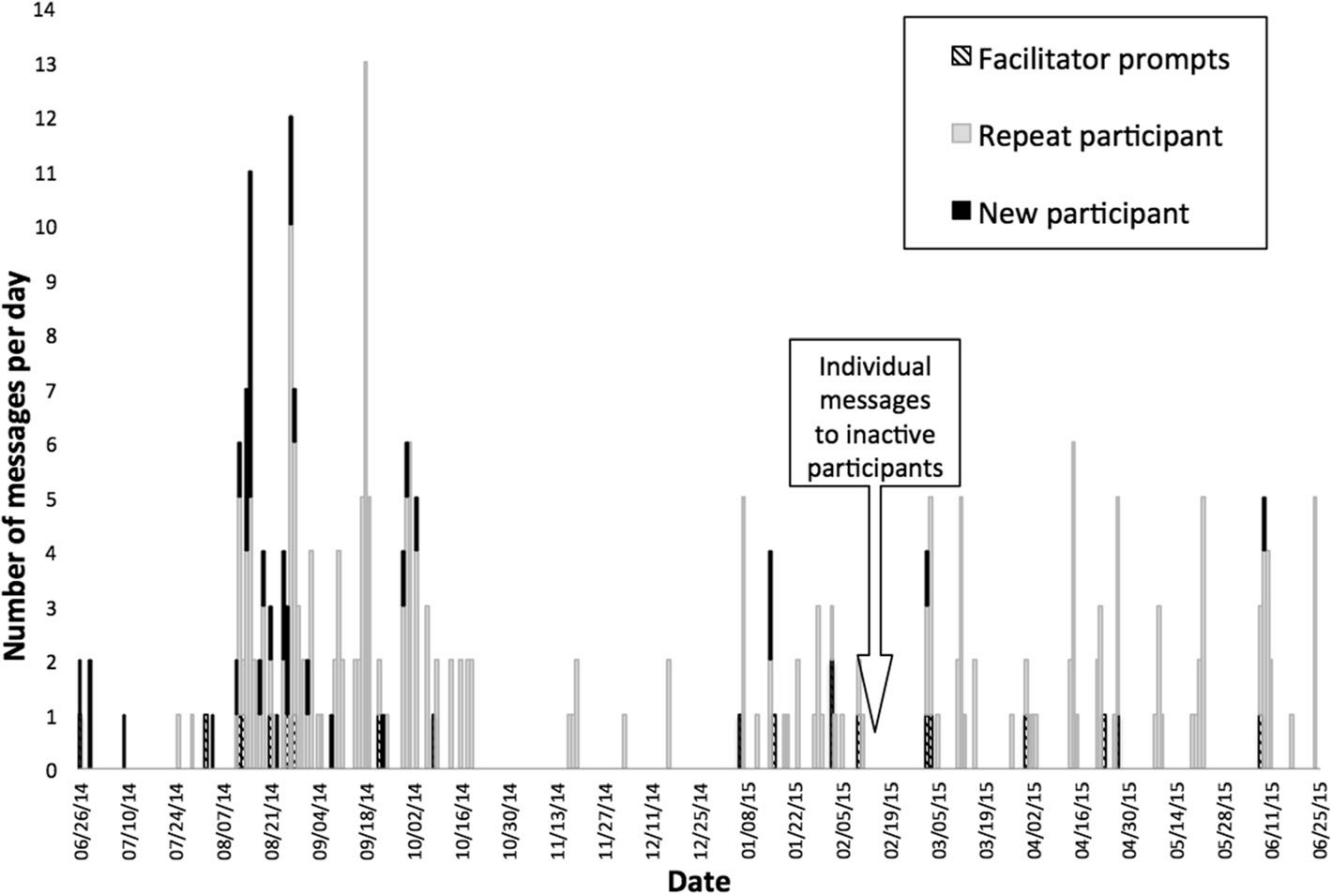
Chronological frequencies of daily activity on the group email listserv (*n* = 46, 35 participants and 11 facilitators)

Organizer prompts often unleashed flourishes of list activity, including from participants who had not yet contributed, as shown in Fig. 5. In February, just past halfway through the follow-up year, organizers privately contacted the six individuals who had not so far messaged the group, checking in on how things were going and offering assistance. Four of these quiet participants replied individually, and two later posted to the group list. In total, 94% of all 35 participants were active in online “e-mentoring,” either individually or on the group list.

##### Discussions were relevant and helpful

Figure 5 shows that leaders frequently prompted the list; the timing and content of these prompts were important. List activity was concentrated in the fall, early in the academic year after the summer workshop, as many instructors prepared and began teaching their first IBL class. Leaders prompted the list with relevant topics, asking participants to share their first-day plans, offering advice, and checking in on how first-day activities went. The listserv went relatively quiet again until another, smaller burst of activity in January. Again, organizers provided relevant prompts, asking participants to reflect on what went well (or not) during their first IBL course and querying their plans for the upcoming term. For the rest of the year, activity came in short bursts of responses over a day or two as people responded to a specific question or announcement. On follow-up surveys, most respondents said the list was a “great help” (39%) or “much help” (22%), and none reported that it was “no help.”

These analyses revealed some useful findings about how the email listserv functioned but did not capture other elements we saw in these messages. For example, it seemed that over time, participants became more likely to initiate new exchanges. It also seemed that, although frequency analyses showed that most participants only sent a handful of messages and some waited a long time, the collective discussions helped even those not actively participating. For example, a few “new participants” who contributed only late in the year offered long, thoughtful reflections that referenced earlier discussions, suggesting they had been actively tracking and mentally engaging in the online cohort even though they had not previously posted. We used SNA to analyze the patterns of conversation and test these hypotheses.

### Social network analysis results

**Fig. 6:**
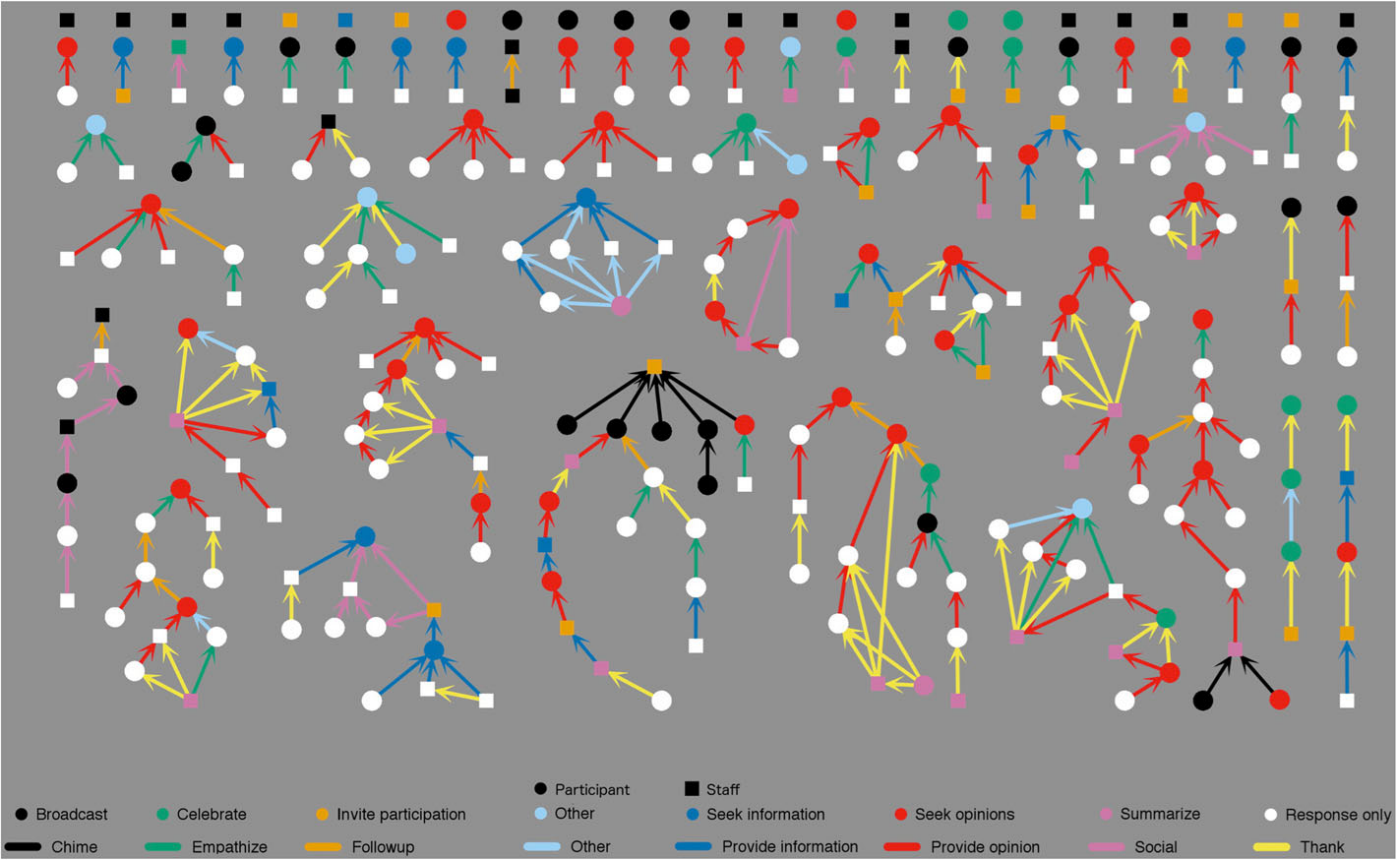
Network diagram of connection between 281 messages sent on listserv in year following workshop, all coded

**Fig. 7:**
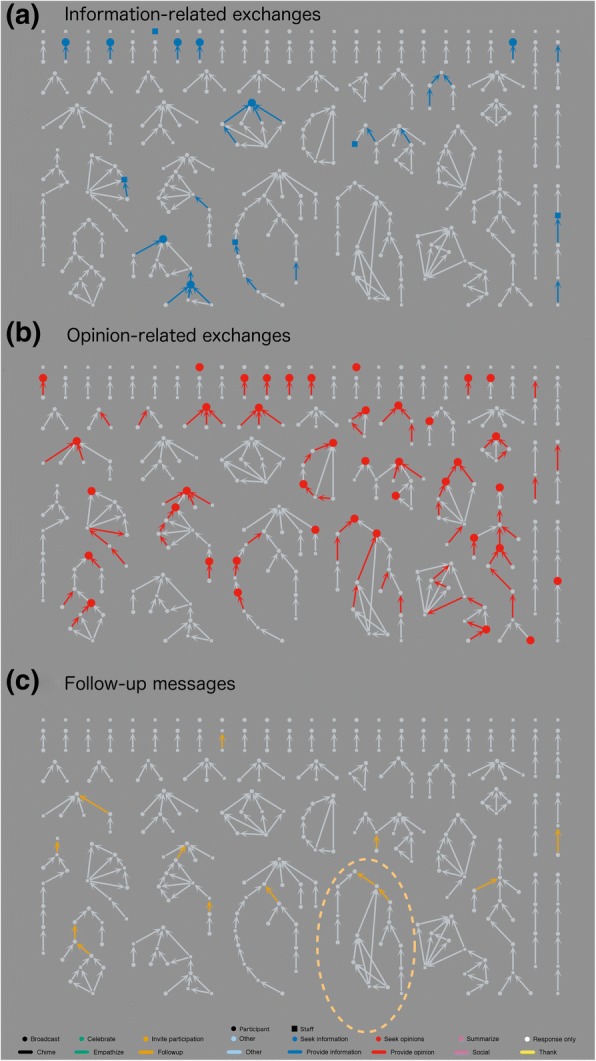
Network diagrams highlighting **a** information*-*related exchanges, **b** opinion-related exchanges, and **c** “follow-up” messages

After coding all of the *message* (forward, generative) and *connect* (backwards, responsive) *functions* as described (Fig. 2), we generated a network diagram. This analysis only includes the 281 messages exchanged in the group listserv and does not account for any private conversations among participants and/or leaders. Figure 6 shows the complete coded diagram. In the following sections, we report results identified from patterns in the network diagram. While each of these patterns is present in the full network of Figs. 6 and 7a–c, each highlight only one relevant function so that patterns stand out. These are intended to help readers more easily visualize results discussed throughout the paper and to make patterns more visible when viewed in greyscale. Additional file 1 contains a more detailed explanation of how to interpret the data presented in message network figures.

**Fig. 8:**
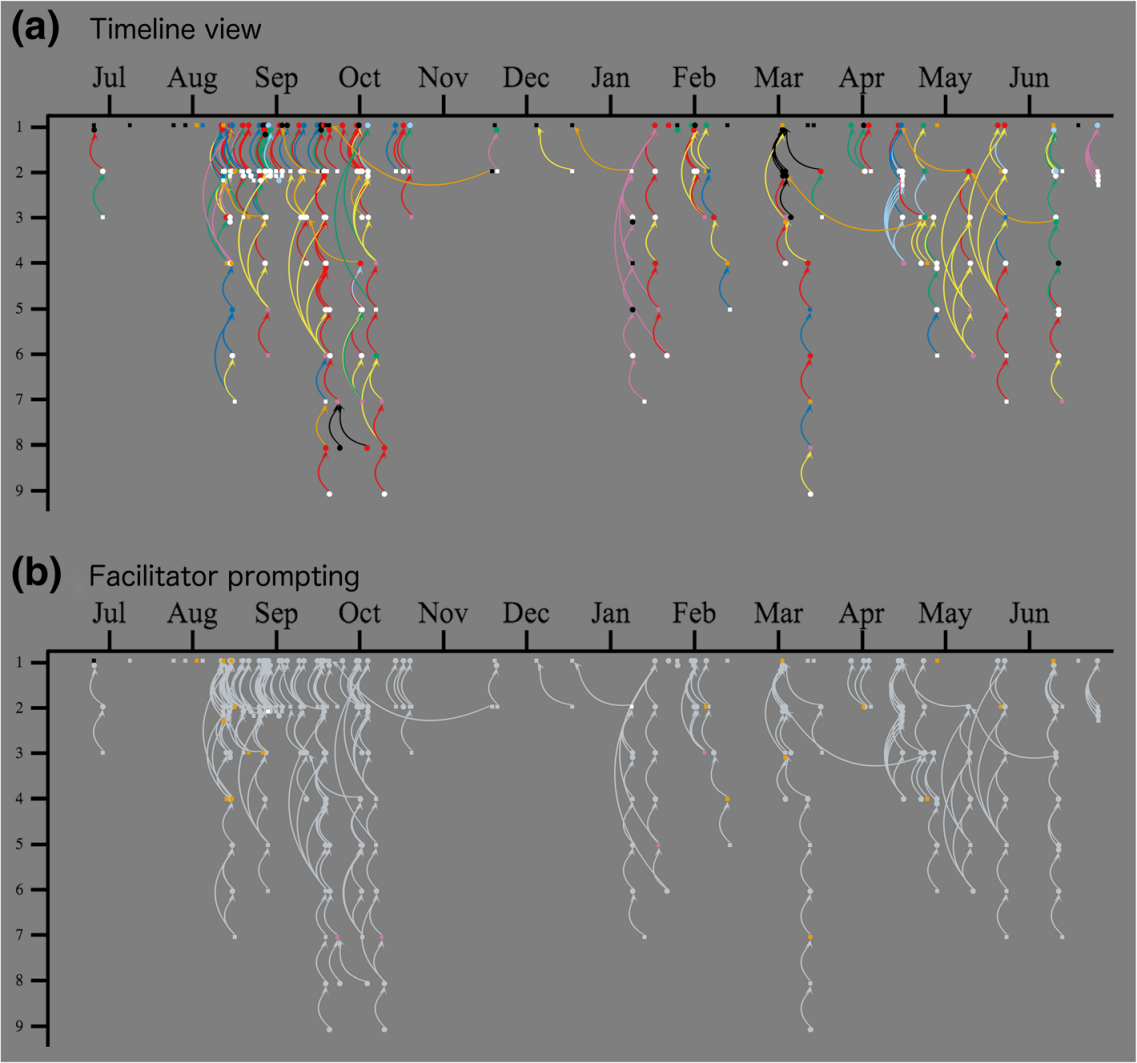
**a** Timeline views of message networks, organized by level in conversation thread with **b** facilitator prompting highlighted

**Fig. 9:**
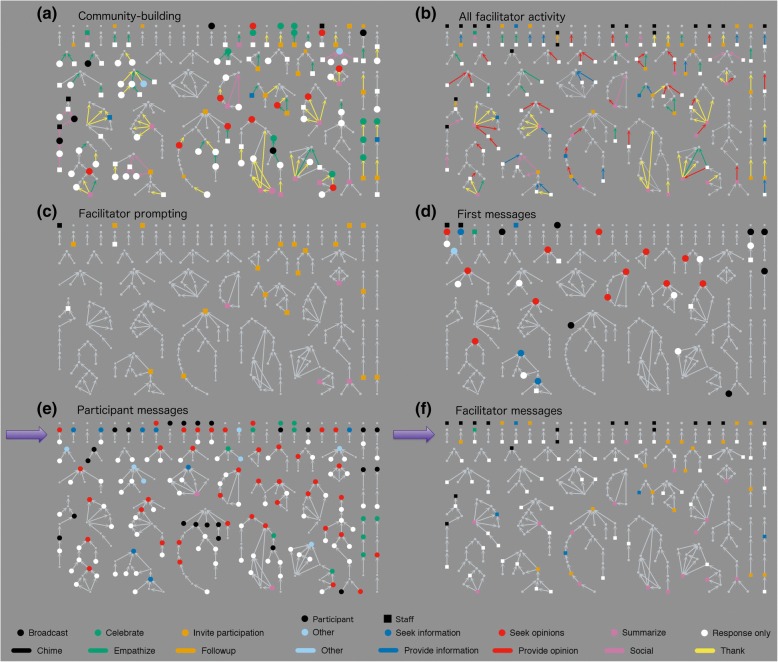
**a**–**f** Network diagrams highlighting ancillary functions and sender characteristics

In all figures, round nodes represent messages from workshop participants and square nodes represent messages from leaders. Each conversation thread is represented as a cluster of connected messages. Nodes are generally arranged by conversation, with earlier messages at the top and response messages below, similar to a genealogy tree. (Figs. 6–9) use a common layout that focuses on conversational complexity: messages with no response are placed at the top of the figures, messages with one response below that, and more complex conversations with multiple branches arranged below these, with increasing complexity from upper left to lower right. The layouts of some conversational clusters in the figures were adjusted slightly for efficient use of space and so that conversational patterns are evident. Figure 8, on the other hand, uses a time-based arrangement that accurately plots the conversations with no adjustments for visual clarity other than arced edges and a small amount of jitter so that nearby nodes do not overlap. Figure 2 serves as a common display key for all SNA (Figs. 6–9).

To discuss how messages are connected in conversations, we use genealogy terms to emphasize the nested and time-based nature of discussions. Any given message may have a *parent* message(s) to which it directly responds or *child* message(s) that directly respond to it. All messages connected upward from a node in a thread are *ancestors*, and all messages connected downward are *descendants.* The *reach* of each message measures the size of its cluster or “family” as the maximum number of other messages connected in the thread in any direction. Some messages are *isolates* with no connections and a reach of zero.

For each message in the network, we calculated the number of parent, child, ancestor, and descendant nodes, and the reach. These values reveal patterns in conversations. For example, message types that more often generate new conversations have many descendants. Conversely, messages that occur later in conversations, like “summarize” messages, have many ancestors. These metrics are presented in Table 1. Confidence markers indicate if the averages for each type vary significantly from the average for the entire network. In the following sections, we share findings from these analyses.

**Table 1:**
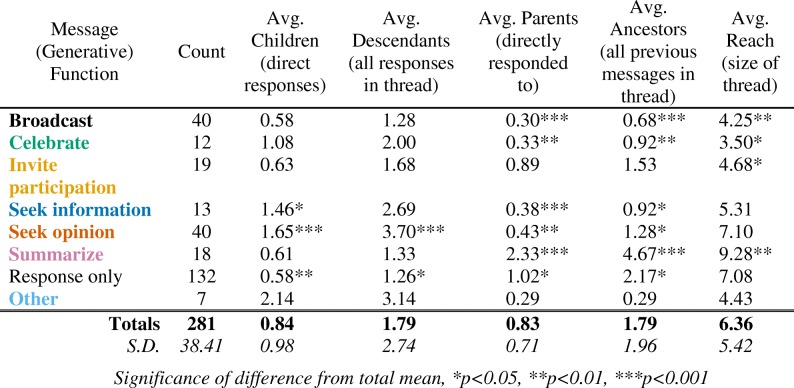
Network metrics by message type

#### The listserv was interactive, not just one-way dissemination

While listservs enable leaders to disseminate information to list members, that was not a primary function of this list. Only 40 of 281 messages (14%) were “broadcast” messages, meaning individuals were disseminating information to the list. Moreover, facilitators sent only 17 of these “broadcast” messages, so only 6% of all list activity was facilitators disseminating information such as upcoming conferences, events, or resources of interest. Their low reach (*M =* 4.25, *t*(39) = − 2.46, *p* < 0.01) means they are not likely to contribute to active conversation threads. In fact, most “broadcast” messages received no responses (21 of 40, 53%), and many of them are isolates (15 of 40, 38%) with no inbound or outbound connections.

#### Conversations were generated and sustained through questions

Questions and answers sparked many messages and more complex threads. Both information- and opinion-seeking messages tended to start discussions, as evidenced by their low average numbers of parent messages (*M =* 0.38, *U* = 1086.5, *p* < 0.001 and *M* = 0.43, *t*(39) = − 3.61, *p* < 0.001, respectively). While both types of messages yielded direct responses (child messages), opinion-seeking messages more often generated rich threads, as evidenced by the high number of descendant messages (*M =* 3.70, *t*(39) = 4.41, p < 0.001).

Figure 7a highlights information-related messages and connections. Conversations were started by an information-seeking message when participants searched for specific resources: for example, course notes or textbooks, tools such as group-creating software, scoring rubrics, or videos or articles about IBL. In ongoing conversations, information-seeking messages usually followed a check-in or request for help and sought more information about the course, the student audience, or strategies the instructor had already tried. Figure 7b highlights opinion-related messages and connections. Typical topics included strategies for increasing student engagement during group work or for encouraging students to present work on the board, ideas for using IBL in larger classes, and systems for grading active-learning components such as presentations or group work. Information- and opinion-related messages are differentiated by whether they were seeing specific answers (information) or were inviting conversation (opinion).

“Follow-up” messages were particularly important for sustaining conversations. These messages were sent in reply to an earlier conversation after some time had passed, usually after the group had discussed a particular problem that one individual was trying to solve. A “follow-up” message then lets the group know what had happened as a result of the earlier discussion. Thus, “follow-up” connections tended to revive the discussion and create new sub-threads; Fig. 7c highlights the connections coded as “follow-up” and shows how they often initiate a new branch of conversation. The example conversation circled in Fig. 7c is described below.

Figure 8a represents the same message networks on a timeline to highlight the time-based nature of conversations. Here the vertical axis represents the sequencing of the conversation thread: discussion starters at the first level, first responses below them, responses to responses at level 3, and so on. This figure shows that most conversations happen over a day or two (81% of responses occurred within 2 days of the original message), as evidenced by the largely straight vertical conversations. (To reduce overlap, arrows are arced and messages in the same level are displayed with a small amount of added jitter.) “Follow-up” arrows tend to go backwards in time to respond to much earlier conversations; the long branches connected below some show renewed discussions. Most striking in Fig. 8 (and the circled conversation of Fig. 7c) is the opinion-seeking message from mid-April that initially received three replies but was revived twice—once in early May with four messages and again in early June with five more messages. This thread started when an instructor shared plans for an upcoming course and got some feedback. Over a month later, the instructor requested and received additional help with group homework assignments that were not going as well as hoped. A month after that, the instructor shared that he was “unbelievably thrilled” with how well the course had gone and thanked the group for their support, which he cited as key to the success of the course.

#### Facilitators purposely built a supportive online community

Of the 281 messages, the main message or connect functions of 88 of them (31%) were coded with the community-building codes “celebrate,” “emotional support,” “thank,” or “friendship.” “Celebrations” included successes in class and personal events such as the birth of a child. “Friendship” included activities not related to instruction, such as making dinner plans at a professional meeting or discussing the outcome of the World Cup, since many had watched early games together at the workshop. “Emotional support” was offered to participants who explicitly said they were struggling with aspects of teaching or professional life. At other times, it was offered to those seeking help with course design, as others chimed in to confirm the difficulty of what they were trying to do. Responses like this early in the year helped to establish a norm of sharing emotions and difficulties with the group, since others offered support and encouragement in response. Overall, group conversations were candid and open, perhaps surprisingly so for a professional cohort.

Forty-five other messages (16%) also set a positive tone by expressing thanks, support, a desire to reconnect, or congratulations, though community building was not the primary coded function. Together, almost half of all messages (133, 47%) included some element of community building as a primary or ancillary function. Figure 9 highlights ancillary functions and sender characteristics; all messages and connections with community-building functions are in panel a. Figure 9b highlights all messages and connections sent by facilitators (participant messages and connections are grayed out). Facilitators sent numerous “summarize” messages with multiple “thank” connections. Indeed, “summarize” messages come almost exclusively from facilitators, often towards the end of a conversation thread, as evidenced by the high number of parent (*M* = 2.33, *U* = 772, *p* < 0.001) and ancestor messages (*M =* 4.67, *U* = 568, *p* < 0.001) compared to descendant messages (*M* = 1.33, *U* = 2288.5, *p* = n.s.). These types of messages do not create new conversation but tend to provide closure and maintain a positive, helpful email community. For example, one such message capped an ongoing discussion on grading group worksheets. The facilitator thanked the participants who had provided opinions and summarized how various decisions would affect student engagement. This provided context for each choice and made it easier for participants to pick an option that would align with their goals. The similarities between Fig. 9a, *Community-building*, and Fig. 9b, *Facilitator activity*, highlight that much of the community building is done by the facilitators and conversely, much of the activity of facilitators is community building.

#### Facilitators made sure the list was helpful

From our discussions with workshop facilitators, we know that they were thoughtful in taking active roles to make the list a useful resource for participants. These roles are evident in the network data.

First, facilitators actively encouraged participants to use the listserv, as seen in the bursts of list activity when facilitators sent prompts (Fig. 5). Figure 9c reveals more details about how the prompts worked, showing all messages with a primary or ancillary *message function* of “invite participation.” Most prompting messages (58%, 15 of 26) did not yield further discussion, and only 23% (6 of 26) have multiple descendants. While facilitators often invited participants to “chime” or “check in,” only eight messages were coded as “chimes,” meaning they did only what facilitators asked by responding with a simple update on their courses.

However, it is clear that facilitators’ persistent prompting established an expectation that participants would engage in the list. Many participants referred to “chiming in” when starting new threads or when responding to messages other than facilitator prompts. For example, 23 participant messages coded as “broadcast” also shared an update on the sender’s course; others provided an update in messages coded as “seeking opinions” or “seeking information.” One participant started a new thread with an “overdue check-in” giving a thoughtful account of two sections of his course and asking for insights into why these similarly taught sections gave quite different outcomes. This message, coded “seek opinion,” initiated an active discussion of 10 other messages, seen as the thread in the lower left corner of Fig. 6. There were numerous other messages that also showed that participants had internalized facilitators’ expectations through the use of common prompting phrases—“chiming in,” “checking in,” or “providing an update.”

Facilitators built these expectations through consistent invitations. The timeline (Fig. 8b) shows that almost half of facilitator prompts (46%) occurred in the first 3 months after the workshop. Later prompts served as reminders to keep participation levels high. Facilitators crafted “low floor, high ceiling” prompts that were easy for participants to respond to (low floor) but allowed for more complex discussions (high ceiling). Low floor prompts encouraged participants to check in and describe what they were doing, rather than asking for evaluative reflections that might be more intimidating, especially for participants struggling with IBL. Yet, these simple check-ins often generated rich discussion (high ceiling). The example message just previous is a perfect example: an observation about the difference between two sections (low floor) started one of the richer discussion threads on the listserv (high ceiling). Thus, persistent prompting and inviting generated many conversations indirectly.

Second, once this list culture was established, facilitators were careful with the timing of their own responses. They let participant questions sit for a day or two to create space for other participants to respond first; they felt weighing in with an “expert” opinion too quickly would end conversations. This bears out somewhat in the data; when facilitators provided opinions, further discussion from participants occurred only 33% (11 of 33) of the time, but when participants provided opinions, 43% (18 of 42) led to further participant discussion. By not jumping in too quickly, facilitators helped to transfer authority to the group over time. As a result, participants tended to serve as resources for each other: 56% of the 75 messages providing opinions were from participants. Facilitators’ frequent thanks to participants who engaged in discussions also encouraged more participant engagement.

Moreover, although facilitators waited before responding, they were careful to not let participant questions go unanswered. Response times were generally quick and did not vary much: of the 235 response connections in the dataset, most occurred within 1 (69%) or 2 (81%) days of the initial message. Only 15 responses (6%) occurred more than a week after the original message and half of those (53%) were *follow-up* messages. There was a weak correlation, *r*(235) = 0.15*, p* < 0.05, between length of follow-up and number of descendant messages, but this is skewed by long-term follow-up messages that tended to revive rich conversations with new information.

In general, facilitators “struck while the iron was hot” and tried to make sure messages had at least one response within 2 days. Of the isolate messages at the top of Fig. 6, only two of 24 are unanswered participant messages seeking opinions. This contrasts with the row below of single-response threads, where facilitators often replied (64%, 14 of 22) so that questions did not go unanswered. Figure 9e, f distinguishes participant and facilitator messages to make this pattern clearer.

By establishing this norm that questions would not be ignored, the listserv became a helpful resource for participants. In fact, 15 of the 31 active participants (48%) sent their first message to the group to seek information or opinions (Fig. 9a), and participants started almost all (23, 88%) of the 33 discussion threads that had more than one response. Moreover, six participants (19%) explicitly stated that they had been “lurking” and found the ongoing discussions helpful even before they joined the conversation.

## Discussion

Evidence from both the follow-up surveys and activity on the group listserv indicates that the workshop was largely successful in helping instructors through *changing* by beginning to use IBL practices in their classes in the year following the workshop. This analysis focuses on the third and final stage of Paulsen and Feldman’s (1995) theory of instructor change, *refreezing*, because it often presents the biggest challenges: support and positive feedback during this time are crucial so that participants do not return to their original strategies (Connolly and Millar 2006)*.* Feedback and support may be most convenient when available at an instructor’s home institution, but only about 43% of participants described such active support from colleagues. While support is essential during *refreezing*, more than half of survey respondents reported no active, local support.

Workshop participants and organizers can serve as a different source of support for instructional change. To overcome geographic distance, the workshop organizers used several methods to support participants through the *refreezing* stage. Participants took advantage of in-person IBL-related events and resources, but the group email listserv was the most widely used support. Both survey responses and participants’ comments in emails indicate that the listserv was helpful.

Many listservs serve as a one-way tool for leaders or moderators to disseminate information to the wider group. Instead, this listserv fostered a multi-dimensional conversation network. Information-disseminating or “broadcast” messages on this list did not often generate responses, but when participants sought information or opinions to help with challenges they were facing, rich discussions often followed. Moreover, we found that, in cases when participants responded to each other, this led to further conversation more often than in cases when facilitators were the first to respond.

According to Paulsen and Feldman (1995), successful *refreezing* depends on intellectual and emotional support and positive feedback. The network analysis showed that participants received and gave emotional support, as about half of messages had an emotional component to them. The connections between opinion- and information-related messages also show evidence of intellectual support—when participants asked questions, they got appropriate answers. Participants used the list to get intellectual support by finding resources, refining their teaching methods, and working through challenges.

The last component, positive feedback, appeared as we followed the list throughout the year. In multiple examples, participants asked a question, received some replies, tested those ideas, and reported back to the group. Indeed, a handful of participants explicitly said that the support they received on the group listserv made their IBL classes successful. Network analysis models these connected threads, including the critical “follow-up” connections, and provides evidence that the workshop listserv provided crucial positive feedback during the refreezing stage. We have only looked at participants’ initial attempts to use IBL in the year following the workshop, and it is possible that they may return to their original methods rather than continuing to use IBL. However, Lewin (1947) found that changes are larger and more lasting when initiated following participatory group discussions compared to lectures or individual decisions. This suggests these initial changes to use IBL may persist. Ongoing studies from our research group will analyze the longer-term trends of instructional change following IBL workshops.

Overall, our findings are similar to those reported by Vaughn and Garrison (2006), who saw shifts in online communications over the course of a PD program. In their study, participants’ social interactions started by sharing emotions and building trust but then shifted to encouraging collaboration over time. Like our study, they also saw a shift in cognitive interactions from simply exchanging ideas to connecting ideas. The online and in-person components in Vaughn and Garrison’s study were concurrent, whereas in our study, the online interactions followed the in-person workshop. Compared to their qualitative approach, using email messages as both a support mechanism and data source may be less resource-intensive and more feasible for researchers and evaluators.

Our findings show why this method of online support was successful at establishing a productive space where participants could interact to find resources, troubleshoot, and improve their teaching. The findings offer some lessons for those looking to support instructors involved in changing their teaching practices. Online communication can enable instructors to interact with like-minded, supportive colleagues, especially if they have none at their home institutions. At least at the time of this study, it seems that listservs or other “push” technologies that direct messages to participants make it more likely that participants will engage, in comparison to online forums that require participants to actively seek out information and help. Listservs are also relatively inexpensive and easy to set up and manage. This listserv was successful because facilitators provided prompts that encouraged participation and kept discussions relevant, in effect enabling participants to give each other “just-in-time” implementation support as they tried new teaching strategies. Rather than solely offering online mentoring by experienced instructors, the listserv became an interactive community where peers supported each other.

Applying social network analysis to these email messages provided a powerful way to model the support processes happening on the listserv. Through SNA, we were able to test hypotheses we had formed about the relationships between different types of messages in ways not possible using different methods that are not focused on relationships, such as frequency analysis.

## Limitations

While in this study the use of social network analysis provided insights not possible through other means, SNA is not always an appropriate tool. SNA has a steep learning curve and is only suited for data that is relational and in a clearly defined network whose complexity can be modeled by only a few categories (Düring 2015). In our case, standard frequency analyses did not capture the complex relationships among email messages. We categorized messages and connections through manual qualitative coding of email messages. These coded data were then a good fit for social network analysis, because of the relation-focused data, clear bounds to the network, and high participation of cohort members. Researchers should be careful to not be seduced by the power and visual appeal of SNA but instead should carefully consider the appropriateness of the methods for each context (Butts 2009). Researchers must also make thoughtful choices when coding and visualizing so that meaningful signals are not overwhelmed by non-essential yet colorful and visually appealing noise. For example, viewing our network in the standard manner using people as nodes (Fig. 1) was not informative, but treating the messages as nodes and pairing qualitative coding of message and connection types allowed for a deep, illuminating analysis of conversational patterns.

As Düring’s “Should I do network analysis?” flowchart (2015) indicates, even with relational data that is sufficiently complex and can be modeled with only a few categories, analysts must really know the data well. For us, qualitative coding required deep understanding of the email messages that we had formed as members of the listserv. This was important because decisions made in coding have a large effect on the resulting networks. For example, social functions could have been overlooked, as participants would often exchange social pleasantries while also providing an opinion. We worked around this barrier by separately coding ancillary “community-building” functions for each message, independently of primary *message* and *connect functions*. We also coded “facilitator prompting” independently of primary *message* and *connect functions*, since we knew this prompting often happened within other types of messages.

The structure of message networks is also affected by coding decisions. Sometimes, messages explicitly refer back to previous messages by name and it is easy to code the connections. When these references were not explicit, the connections were difficult to code. In these cases, when a non-specific message continued an ongoing thread, we connected it only to the most recent message in the thread. In other cases, when a message brought up a new idea related to the topic, we started a new branch from the original message. Such coding is painstaking, as the coder must reread prior messages, sometimes multiple times, and look at quoted replies.

We also carefully chose the metrics we used to analyze the networks. Network-wide metrics like centrality or density did not make sense in this context, since conversations form many separate sub-networks rather than a single interconnected network. Our node-level metrics of “parent” and “child” messages essentially measure out-degree and in-degree connections, yet help to better communicate the sequential nature of the connections. Though “reach” is similar to the more common distance metric, reach is a node-level measure appropriate for the many sub-networks in these data, while distance is a network-focused metric more appropriate for a single, interconnected network. By modifying our terms, metrics, and layout of our diagrams, we could better reflect the specific parameters of this network as both time-based and comprised of distinct conversational sub-networks—contrasting with other applications of SNA that examine one large, interconnected network where the sequence of connections is not as important. While SNA is a powerful method, it is not appropriate in many situations; in others, it may demand significant investment in qualitative coding and technical modifications to better fit the context. However, in the right context, social network analysis allows the analyst(s) to examine relationships between actors and to model processes as they develop over time.

## Conclusions

This study offers evidence of successful online support through a group listserv following a professional development workshop on teaching for college mathematics instructors. Although online follow-up support is a common practice, it has not been well studied, and what is available tells us little more than the frequency of interaction. By applying the techniques of social network analysis paired with qualitative coding of email messages, we saw evidence of intellectual and emotional support, as well as productive feedback loops. Moreover, though many PD workshops provide online support through group listservs, it is often difficult to get participants to engage in them. Our analysis revealed how these facilitators created an engaging, productive listserv, and this example may help other professional developers to create more productive listservs or enable other scholars to study listserv function.

## Additional file

Additional file 1: Interpreting message network figures. High resolution versions of all figures are available from the authors. (DOCX 126 kb)

## Abbreviations

AIBL: Academy of Inquiry Based Learning
IBL: Inquiry-based learning
JMM: Joint Mathematics Meetings
NSF: National Science Foundation
PD: Professional development
SNA: Social network analysis
STEM: Science, technology, engineering, and mathematics
