# Supplementary material for: Supporting instructional change in mathematics: using social network analysis to understand online support processes following professional development workshops
Source: Int J STEM Educ. 2018 Jul 2;5(1):28. doi: 10.1186/s40594-018-0120-9 (PMC6310432; doi:10.1186/s40594-018-0120-9)
Supplement: Supplementary file 1 — Interpreting message network figures. High resolution versions of all figures are available from the authors. (DOCX 126 kb) [file 40594_2018_120_MOESM1_ESM.docx]

**Additional file 1: Interpreting Message Network Figures.**

Figure S1. Simple example of network diagram.


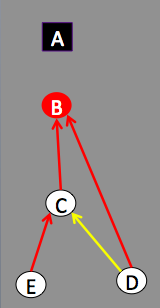


In all figures, round nodes represent messages from workshop participants and square nodes represent messages from facilitators. Arrows represent the direction of the responses between messages. For example, if message B responds to message A, the arrow points from B to A. Since network diagrams communicate lots of information in a compact manner, Additional file 1 Figure S1 is a simple example to illustrate how to interpret these diagrams and the networks of messages they represent. Node A represents a *broadcast* message (black fill color) from a staff member (square shape) that received zero responses (no connected arrows). Node B represents a participant message (circle) that is *seeking information* (red) and received two responses which *provided information* (the two incoming red arrows from C and D). Node C is a participant message (circle) that was a *response only* (white) that *provided information* to the question posed in message B (red arrow from C to B). Node D was also a participant (circle) *response only* message (white) that *provided information* responding to message B (red arrow) and also thanked the response from message C (yellow arrow). While it may sound strange to say “thanked the response from C,” this means that the participant who sent message D thanked the participant who provided the response in message C. As this example shows, when the nodes represent messages, conversations and topics develop into threads. A more traditional network diagram where the nodes represent people, rather than messages, only tells us who is talking to whom.

In network figures, nodes are generally arranged so that earlier messages are at the top, and response messages are below that, similar to a genealogy tree. To discuss how messages are connected in conversations, we use terms from genealogy, emphasizing the nested and time-based nature of discussions. For example, if Node C is the reference node, it has one *parent* message (B), which is one link above it, and two *child messages* (C and D), which are one link below it. All previous messages are *ancestors* and all following messages are *descendants.* These values are always calculated in relation to a reference node, and can be calculated for each node in the networks. For example, Node B has three descendants (C, D, and E) but no ancestors. In contrast, Nodes D and E both have two ancestors (B and C) but no descendants. While both Nodes E and D have two ancestors, they differ because E has one parent message while C has two parent messages. The *reach* of a message means the number of other messages to which it is connected in the network. For example, Nodes B, C, D, and E all have a reach of three since from any of them, there are paths to three other nodes. Essentially, the *reach* is the size of the “family.” The genealogy language does not always hold, as some nodes have no connections, or a reach of zero, such as A, and are called *isolates*.
